# 4H-SiC PIN Diodes as Environment to Modify ^7^Be Radioactive Decay Time

**DOI:** 10.3390/ma19132741

**Published:** 2026-06-26

**Authors:** Virginia Boldrini, Luigi Di Benedetto, Vincenzo Carrano, Mariaconcetta Canino, Nicola Casali, Raffaele Buompane, Claudio Santonastaso, Maria Lucia Mitsou, Kajol Chakraborty, Ravi Prakash Yadav, Arpana Singh, Marco Pieruccini, Cristian Degli Esposti Boschi, Matthias Laubenstein, Alfredo Rubino, Alba Formicola, Heinz Christoph Neitzert, Lucio Gialanella

**Affiliations:** 1 Istituto per lo Studio dei Materiali Nanostrutturati (ISMN), Consiglio Nazionale delle Ricerche (CNR), Via Piero Gobetti 101, 40129 Bologna, Italy; virginia.boldrini@cnr.it (V.B.); marco.pieruccini@cnr.it (M.P.); cristian.degliespostiboschi@cnr.it (C.D.E.B.); 2 Dipartimento Ingegneria Industriale, Università degli Studi di Salerno, Via Giovanni Paolo II 132, 84084 Fisciano, Italy; ldibenedetto@unisa.it (L.D.B.); vcarrano@unisa.it (V.C.); asingh@unisa.it (A.S.); arubino@unisa.it (A.R.); neitzert@unisa.it (H.C.N.); 3 INFN Sezione di Roma, P. le Aldo Moro 2 c/o Dipartimento di Fisica “Sapienza” Università di Roma, 00185 Roma, Italy; nicola.casali@roma1.infn.it (N.C.); ravi.prakash.yadav@roma1.infn.it (R.P.Y.); alba.formicola@roma1.infn.it (A.F.); 4 Dipartimento Matematica e Fisica, Università della Campania “L. Vanvitelli”, Viale Lincoln 5, 81100 Caserta, Italy; raffaele.buompane@unicampania.it (R.B.); claudio.santonastaso@riken.jp (C.S.); marialucia.mitsou@unicampania.it (M.L.M.); kajol.chakraborty@unicampania.it (K.C.); lucio.gialanella@unicampania.it (L.G.); 5 INFN Sezione di Napoli, Complesso Universitario di Monte S. Angelo ed. 6 Via Cintia, 80126 Napoli, Italy; 6 INFN Laboratori Nazionali del Gran Sasso, Via G. Acitelli 22, 67100 Assergi, Italy; matthias.laubenstein@lngs.infn.it

**Keywords:** 4H-SiC, PIN diodes, reverse bias, Be ion implantation, capacitance

## Abstract

This work explores the possibility of using 4H-SiC PIN diodes to provide a high electric field able to induce the Stark effect in ^7^Be atoms implanted in the diode space charge region, modifying the ^7^Be radioactive decay time. A set of PIN diodes of area ranging between 2.12 × 10^−3^ cm^2^ and 9.88 × 10^−3^ cm^2^ was designed and fabricated to reach breakdown voltages up to 1000 V. Be ions were implanted in the epitaxial layer, and then the devices were reverse biased at about 75% of the theoretical breakdown voltage for durations exceeding 100 days, long enough for a precise measurement of the ^7^Be radioactive decay time. Electrical characterization in the pristine state, after Be ion implantation, and after long reverse bias allowed us to verify the suitability of 4H-SiC PIN diodes by assessing both the agreement between simulated and measured performance and the stability of the electric field. Be ion implantation-related defects induced both an increase in the reverse current generation and a decrease in the junction capacitance, though not affecting the breakdown voltage. Comparison with test devices implanted with the stable isotope ^9^Be indicates that any defects introduced by the ^7^Be radioactive decay are below the detection limit of the employed characterization techniques and have a negligible impact on the reverse-blocking characteristics of the diodes. Device simulations allowed us to conclude that the electric field remains close to its theoretical value throughout the experiment duration, confirming the suitability of 4H-SiC diodes for both induction and measurement of ^7^Be lifetime variations.

## 1. Introduction

This work explores a novel application of 4H-Silicon Carbide (4H-SiC), i.e., the use of 4H-SiC diodes to provide the environment to demonstrate the Stark effect in the radioactive decay of ^7^Be atoms. The choice of 4H-SiC is due to its high critical electric field and its good radiation tolerance, features that are widely exploited in power devices and radiation detectors [1,2,3]. Moreover, literature studies on Be ion implantation in SiC [4,5,6] provide the ground for a preliminary assessment of the experiment’s feasibility.

The ^7^Be half-life, that is equal to (53.284 ± 0.016) days in equilibrium conditions [7], is expected to change according to environmental factors such as electric fields, temperature, electronic structure, and host lattice properties, which can typically modify the half-life of ^7^Be by 0.01–1% [8,9,10]. More extreme conditions, such as high pressure, spatial confinement, and tailored molecular systems (e.g., fullerenes), can induce greater variations in electron density near the nucleus and, consequently, in the decay rate. This underscores the key impact of the electronic environment on electron-capture processes [11,12,13,14]. A variation in the ^7^Be half-life might as well be observed when the ^7^Be atoms are in a 4H-SiC junction reverse-biased close to its breakdown voltage. ^7^Be atoms can be inserted by ion implantation inside the wafer epitaxial layer close to the junction, in order to exploit the high electric field that can be attained in the depletion region, of the order of 10^6^ V/cm [15]. In these conditions, implanted ^7^Be atoms bind to locally damaged lattice spots through unsaturated orbitals that were involved in C-Si regular bonding prior to irradiation. Being rather infrequent, the occurrence of substitutional ^7^Be, one can resort to ordinary quantum mechanical methods, such as linear combination of atomic orbitals, to describe the effects of an electric field on the bond orbitals, through the perturbation of the ^7^Be–lattice electric dipole. In the case of a +1 ionized ^7^Be–lattice bond (i.e., with only one of the 2s ^7^Be electrons involved in the bond and the other dispersed in the environment), and also accounting for the randomness of the dipole orientation, we can estimate a ^7^Be life-time decrease in the order of 0.5% for an electric field of 10^6^ V/cm, which in terms of absolute values is in line with the results reported in the literature above; the less stable, non-ionized configuration is expected to be the same order, though possibly of opposite effect on the life-time.

Achieving a measurable change in radioactivity requires implanting a sufficient number of ^7^Be atoms [16]; however, the peak concentration must remain below the donor concentration in the epitaxial layer to avoid severe device degradation due to electrically active defects. A tradeoff can be achieved by performing measurements in underground facilities such as Laboratori Nazionali del Gran Sasso [17], where the low background reduces the activity needed for detection. Additionally, the ^7^Be concentration can be reduced by increasing the diode area and distributing ^7^Be atoms over a wider depth, provided that the electric field variation across the region remains below 20%. An epitaxial layer doping in the range of 10^16^ cm^−3^ responds to these requirements, whereas the practical requirement of maintaining the breakdown voltage around 1000 V limits the epitaxial layer thickness to about 5–6 µm.

A precise measurement of the possible ^7^Be decay time variation can be performed by applying a continuous reverse bias to the PIN diode for a duration comparable to the half-life itself. While standard aging tests of 4H-SiC power diodes comprise application of reverse bias equal to 80% of the breakdown voltage for as long as 1000 h [18], in this work, the biased PIN diode is also subject to 478 keV gamma radiation emitted by the excited ^7^Li atoms, which are the product of the ^7^Be decay.

This work describes the fabrication process sequence of the devices used as a ^7^Be decay environment, with particular emphasis on the effects on the reverse-blocking characteristics induced by ^7^Be ion implantation and ^7^Be decay under high reverse-bias. The objective is to verify the agreement between the electrical behavior of the ^7^Be-implanted diodes and simulation predictions, as well as to assess the stability of the diode characteristics over time. Measurements and analyses of the ^7^Be decay rate are beyond the scope of this work. An electrical characterization of 4H-SiC PIN diodes specifically fabricated for this application was performed: (i) in the pristine state, (ii) after Be ion implantation, (iii) in operando, and (iv) after long-term reverse-bias stress. In the pristine state, current–voltage (I–V) measurements allowed us to select the best-performing diodes. After the Be implantation, I–V characterization was used to optimize the reverse bias to apply during the decay experiment, i.e., to identify a value ensuring a stable reverse current over several days while maintaining a sufficiently high electric field. Device current was controlled during operation and at the end of the experiment. Capacitance–voltage (C–V) characterization of the pristine diodes was used to verify the epitaxial layer doping, which is required for accurate electric-field calculations. C–V curve fitting by Synopsys TCAD Sentaurus [19] simulations allowed for interpreting the effects of defects introduced by ^7^Be ion implantation. Additional analyses on test devices, i.e., Schottky and PIN diodes implanted with the stable isotope ^9^Be, were carried out to independently assess the impact on device performance of damage caused by Be implantation and by the ^7^Be decay.

## 2. Materials and Methods

**Figure 1:**
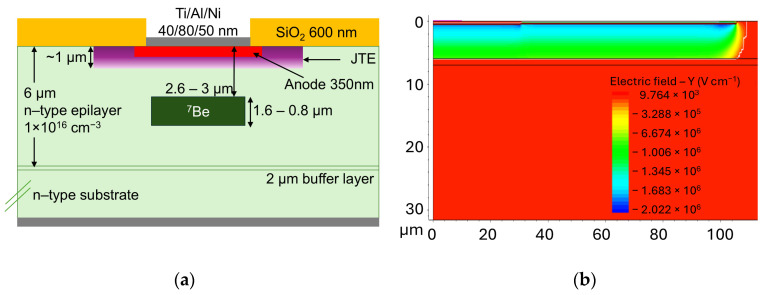
(**a**) Cross section of the PIN diode layout, not in scale; the figure reports the anode and Junction Termination Extension (JTE) depth as well as the depth and extension of the Be implant in D7Be-I and D7Be-II are indicated; (**b**) color scale of the electric field in the epitaxial layer, in the buffer layer, and in the top 22 µm of the substrate calculated by Sentaurus simulation at 750 V reverse bias.

The fabrication process resulted in PIN diodes of different sizes and geometries, as well as test devices such as Schottky diodes, van der Pauw structures, and Transmission Line Model (TLM) structures. The layout of the PIN diodes employed in the ^7^Be decay experiment, labeled D7Be-I and D7Be-II, is illustrated in Figure 1a. Geometrical and doping parameters of the starting wafer, of the anode, and of the junction termination extension (JTE) were determined by Synopsys TCAD Sentaurus simulations in order to obtain the required electric field of 1 MV/cm in the region intended to host Be atoms at 750 V reverse bias, as shown in Figure 1b.

The starting material was an n-type 4H-SiC wafer purchased from Coherent (Saxonburg, PA, USA), with a 6 µm thick 1 × 10^16^ cm^−3^ n-type doped epitaxial layer grown 4° off-axis on a 360 µm thick production-grade substrate, provided with a 2 µm buffer layer. A shallow 5 × 10^19^ cm^−3^ P^+^ ion implantation was first carried out on the wafer back side to improve the ohmic contact. The diode anode and JTE were created by multiple Al^+^ ion implantation carried out at 350 °C through a 1 µm thick SiO_2_ stopping layer, in order to achieve a 280 nm deep plateau with 5 × 10^19^ cm^−3^ Al concentration in the anode region and Al concentration ranging between 3.00 × 10^17^ cm^−3^ and 4.25 × 10^17^ cm^−3^ in the JTE. The post-implantation annealing was carried out on single dies at 1950 °C for 30 min in Ar atmosphere with the 4H-SiC surface coated by a pyrolyzed photoresist cap [20]. After annealing, the cap was removed by dry oxidation at 850 °C. The wafer surface outside the diode area was passivated by a 600 nm thick SiO_2_ layer. The front contact of the PIN diodes was made up of an evaporated stack Ti (40 nm), Al (80 nm), and Ni (50 nm), which constituted also the metal barrier of Schottky diodes realized on the same n-epilayer. 50 nm of Ni was evaporated on the entire back surface. Finally, an alloy treatment was carried out in vacuum at 1100 °C for 5 min.

High-energy ^7^Be ion implantation was performed in selected devices using a double AZ10XT photoresist layer (MicroChem, Newton, MA, USA) as a mask to locate the Be atoms in the diode inner area of 960 µm diameter. The two PIN diodes selected for the ^7^Be decay experiment were subject to ^7^Be multiple implantations in order to form a box profile at depth ranging between 2.6 μm and 4.2 μm and between 3.0 μm and 3.8 μm, respectively. The test PIN diode D9Be underwent ^9^Be ion implantation for a box profile between 1.8 μm and 5.2 μm. A set of Schottky diodes was implanted with 5.1 × 10^12^ cm^−2 9^Be atoms to a 1.75 µm projected range, corresponding to a ^9^Be peak concentration equal to 1 × 10^17^ cm^−3^. Table 1 reports the Be position and concentration calculated by SRIM simulation [21] in the two diodes selected for ^7^Be decay experimentation (D7Be-I and D7Be-II) and the two devices used as test with ^9^Be implantation (D9Be and S9Be). Appendix A describes the Be ion implantation setup, and Table A1 reports the Be energies and the ^7^Li contamination in the ^7^Be implantation beam. The ^7^Li in the ^7^Be beam ranged between 70% and 98% for D7Be-I and between 17% and 21% for D7Be-II.

Current-voltage (I–V) characterization in forward and low reverse bias regimes was carried out using a micromanipulator probe station (Micromanipulator, Carson City, NV, USA) with a thermal chuck and two Source Measure Units, Keithley 238 (Keithley, Solon, OH, USA). The current floor of the whole setup was 1 × 10^−13^ A. High reverse bias I–V measurements, in the [200–1000] V range, were performed using a high voltage power supply combined with a Keithley 487 picoammeter (Keithley, Solon, OH, USA). The high voltage power supply consisted of a 24 V DC continuous power supply (Traco Power TXM050-124) (Traco Power, Baar, Switzerland) with a voltage booster (ISEG, DPS-Series Configuration: 10 106 24 5 ESH) (ISEG, Berlin, Germany), which enabled reverse bias of the SiC diodes up to a voltage of 1000 V with a maximum current of 10 mA. A 10 MΩ series resistance was connected to avoid diode overheating in case of abrupt breakdown. The applied reverse bias has been corrected for the voltage drop over this series resistor. Measurements in the final state are carried out on the diodes bonded to a board.

C–V characteristics were acquired at 100 kHz using an HP4284A precision LCR meter (Keysight, Santa Rosa, CA, USA).

## 3. Results

**Figure 2:**
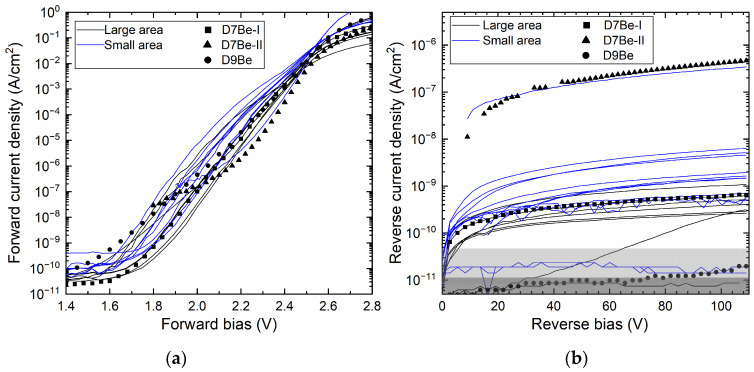
J–V characteristics of pristine devices. The diodes of larger area (anode area equal to 7.85 × 10^−3^ cm^2^ or 9.88 × 10^−3^ cm^2^) are represented in black color: the J–V curves of the three PIN diodes which were selected for further experimentation are highlighted by black symbols, whereas the diodes which did not undergo any further experimentation are represented by black lines. The small area diodes (anode area equal to 1.96 × 10^−3^ cm^2^ or 2.56 × 10^−3^ cm^2^) are represented by blue lines. (**a**) Forward curves; (**b**) Reverse characteristics measured in the [−110 V, 0 V] range. The forward J–V curve of D7Be-II was measured with the same instrument used for high-voltage characterization, which is featured by a higher current floor; therefore, the plot starts at 1.8 V.

An assessment of the pristine device operation was carried out by performing I–V characterization of 40 diodes on 10 different dies in the [−110, +4] V range. Figure 1 shows the J–V curves of the 25 diodes whose −110 V reverse current did not exceed 10 nA. The diodes are divided into two groups according to the size of the p^+^ implanted anode: the large area group is represented by black lines, whereas the small area group is represented by blue lines. Among the large area diodes, 3 devices from 3 different dies were selected for further processing: these are highlighted by full symbols. In Figure 2a, most characteristics show similar slopes in the 2.4–2.6 V range, whereas three curve shapes were identified in the 1.7–2.4 V region: single slope (14 diodes), double slope (4 diodes), and a slope with a bump (7 diodes). Figure 2b shows that at −110 V the reverse current density spans from 10^−11^ A/cm^2^ to 5 × 10^−7^ A/cm^2^ for 21 diodes, whereas for 4 diodes it remains below the detection limit of the measurement setup, indicated by the light- and dark-gray shading.

The order of magnitude of the reverse current is related to the concentration of generation centers in the space charge region, according to the Shockley-Read-Hall (SRH) mechanism. Moreover, it is worth noticing that larger-area diodes exhibit lower current density compared to smaller devices, due to leakage currents originating from the perimeter, whose effect is more pronounced in smaller diodes [22]. Within the probed bias range, which corresponds to a depletion extending 3.4 µm below the junction [23], the reverse current increases gradually, consistent with good rectifying behavior. However, low-voltage characterization might prevent the detection of defects located deeper in the underlying epitaxial layer.

**Figure 3:**
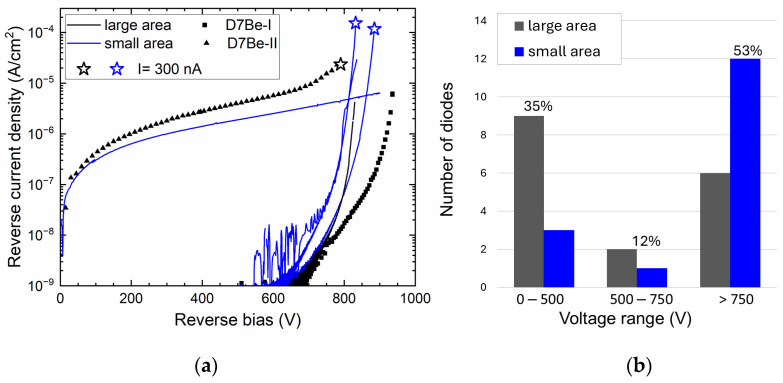
Results from the high reverse bias I–V characterization of the pristine PIN diodes. The black color indicates the large area diodes, whereas the blue color indicates the small area diodes. (**a**) J–V characteristics of the diodes that, on each die, reach the current limit of 300 nA at the highest voltage value. This value is highlighted by a star on the curves where it was reached in the measurement range. The diodes that were selected for the Stark effect generation are highlighted by full symbols. (**b**) Number of diodes capable of sustaining reverse bias in the ranges indicated by the x-axis, with current not exceeding 300 nA, grouped by anode area; the percentages refer to the total of both small and large area devices.

In order to identify the best-performing diodes that can sustain an electric field able to modify the ^7^Be decay, high reverse bias characterization was performed. Figure 3a shows the reverse J–V curves of 7 diodes on 7 different dies, i.e., the diode that, on each die, reaches the current limit at the highest voltage value. Most of the diodes exhibited currents below the detection limit when the reverse bias was lower than 500–600 V, indicating a low defectivity, both native and process-induced, in the epitaxial layer; at higher voltages, the current undergoes a gradual increase up to 700–800 V, due to trap-assisted tunneling (TAT) [24]. A steeper increase occurs at higher voltages, due to avalanche breakdown. Since this characterization aims at the identification of the diodes for further processing and testing, avalanche breakdown attainment was avoided. D7Be-II and one of the small area diodes made an exception with respect to this general behavior, showing a higher SRH current generation in the 0–700 V reverse bias range, followed by a moderate current increase at higher voltages up to the attainment of the current limit, occurring at 800 V (D7Be-II), and above 1000 V (small area diode). Thus, the high SRH generation is not detrimental to the high-voltage blocking. Since the different production batches showed a good repeatability, we attribute the higher SRH current generation to die placement on a more peripheral zone of the wafer. Figure 3b shows a statistic for 33 diodes across 9 different dies, grouped by anode area. The histogram indicates the number of diodes able to sustain reverse bias within the ranges indicated on the *x*-axis while maintaining current below 300 nA. While 35% of the diodes showed premature breakdown (and for this reason were already excluded from the plot in Figure 2b), 55% sustained 750 V with current < 300 nA. On each die, at least one diode was classified as suitable for the experiment, i.e., with a current lower than 300 nA at voltages above 750 V, independently of the diode area. The diodes employed in further processing and testing were selected from the large area group.

**Figure 4:**
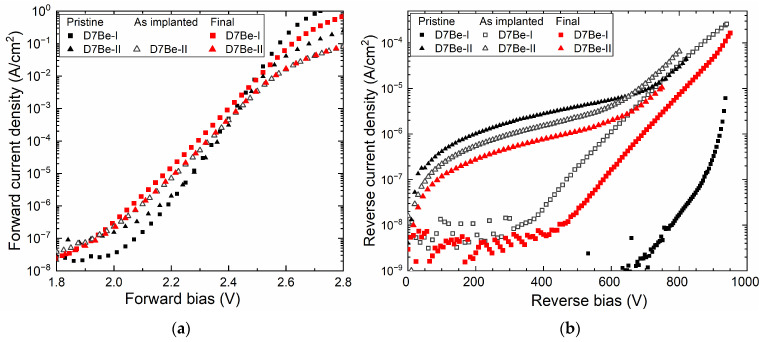
Results from I–V characterization of the two PIN diodes selected for the ^7^Be decay experiment at different experimental stages. The characteristics of D7Be-I and D7Be-II in the pristine state are represented by full black squares and triangles, respectively; the measurements in the as-implanted state are indicated by gray open squares and triangles; the measurements in the final state are indicated by full red squares and triangles, respectively. (**a**) Forward characteristics; (**b**) Reverse characteristics. The J–V curves of D7Be-I are published in [25].

Figure 4 shows the J–V characteristics of D7Be-I and D7Be-II, at different experimental stages, i.e., after ^7^Be ion implantation and after prolonged continuous reverse bias, compared with the J–V curves measured in the pristine state. After ^7^Be ion implantation, the forward characteristic of D7Be-II exhibits a bump, which is maintained after prolonged reverse bias. The forward J–V curve of D7Be-I was not measured in the as-implanted state, in order to start the ^7^Be decay measurements as soon as possible and, therefore, exploit the highest signal from the ^7^Be decay; however, the presence of a bump in the final state, as already observed in the as-implanted and final state of D7Be-II, can indicate the formation of recombination defects at the implantation stage, independent on the shape of the pristine J–V curve. Similarly, Be ion implantation induces an increase in diode series resistance with respect to the pristine state.

The reverse current increases in D7Be-I, while it stays comparable to the pristine state in D7Be-II, which, however, was featured by a higher current in the pristine state. In the final state, i.e., after prolonged reverse bias, the J–V curves of the ^7^Be-implanted diodes show negligible changes. The slight current reduction can be attributed to diode self-healing, as already observed in the literature as a consequence of different phenomena. Leakage current in p+/n 4H-SiC diodes decreased with time at room temperature in [26] or after high reverse bias stress for 10 min in [27] and in SiC diodes, both PIN and Schottky, after electron or He^+^ irradiation [28,29,30]. The reduction was attributed to self-recovery of lattice damage from ion implantation or irradiation, while high reverse bias led to local heating at the SiO_2_/4H-SiC interface, suppressing surface leakage. None of the above occurrences, which are both taking place in this experiment, is expected to determine a variation in the electric field formed in the diode space charge region at high reverse bias, thus an attribution is out of the scope of this work.

**Figure 5:**
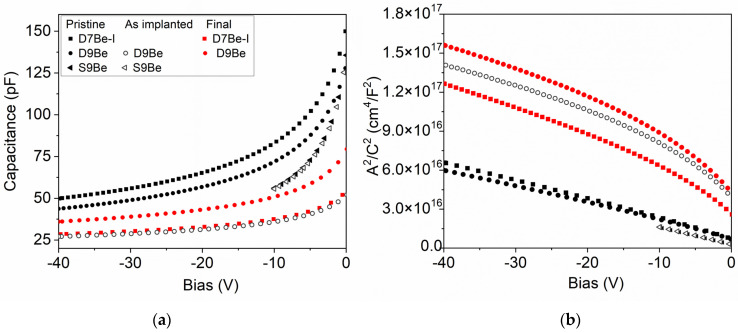

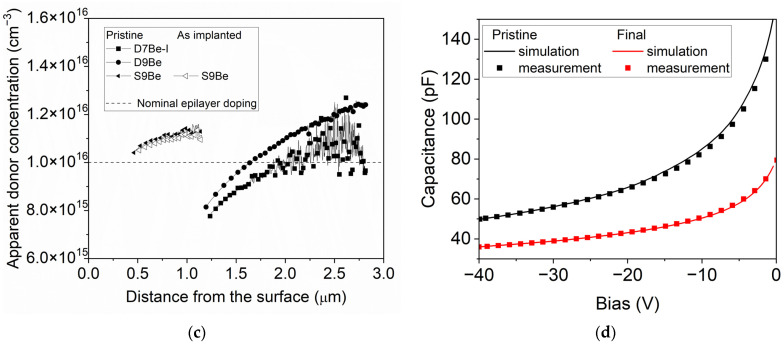
C–V characterization of D7Be-I and of the two test devices D9Be and S9Be at different experimental stages. The curves measured in the pristine state are represented by full black symbols; the measurements in the as-implanted state are indicated by gray open symbols; the measurements in the final state are indicated by full red symbols. (**a**) C–V characteristics of D7Be-I (squares), D9Be (circles) and S9Be rotated triangle in the pristine, as-implanted, and final state; (**b**) A^2^/C^2^ vs. V curves; (**c**) apparent donor concentration extracted from C–V measurements for all the of the devices under test in the pristine state, and, in the as-implanted state, for S9Be only. The profiles of the PIN diodes are shifted by 0.7 µm to account for the depth of the metallurgical junction; (**d**) comparison between numerical simulation results (lines) and experimental C–V curves (symbols) of D7Be-I in the pristine (black) and final (red) state. The C–V curves of D7Be-I are published in [25].

**Figure A1:**
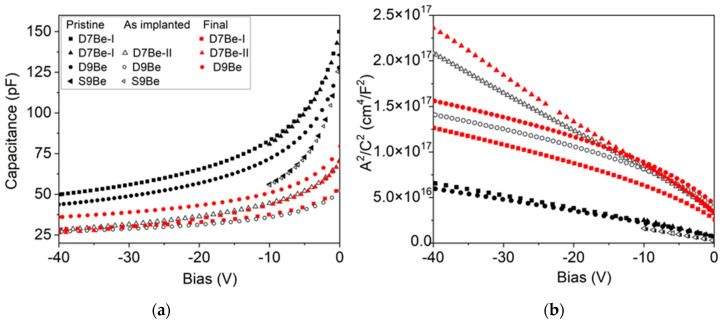
C–V characterization of D7Be-II compared to the characterization reported in Figure 5a,b. The curves measured in the pristine state are represented by full black symbols; the measurements in the as-implanted state are indicated by gray open symbols; the measurements in the final state are indicated by full red symbols. (**a**) C–V characteristics of D7Be-I (squares), D7Be-II (triangles), D9Be (circles), and S9Be rotated triangle in the pristine, as-implanted, and final state; (**b**) A^2^/C^2^ vs. V curves of the devices under test.

Figure 5 summarizes the capacitance–voltage characterization and its interpretation. Figure 5a shows the C–V curves of D7Be-I, D9Be, and S9Be, measured in different conditions: pristine state (all), as-implanted state (D9Be and S9Be), and final state after long-term reverse bias (D7Be-I and D9Be). In pristine condition, all unbiased devices exhibit a capacitance of approximately 130–150 pF (i.e., 11 nF/cm^2^ in all the PIN diodes). After the Be implantation, the capacitance of the PIN diodes decreases to about 40 pF, whereas the Schottky diodes do not show any appreciable variation. Prolonged application of a high reverse bias induces small variations in capacitance. The C–V characterization of D7Be-II yields qualitatively similar results, as shown in Figure A1, which reports the C–V curves and the A^2^/C^2^ vs. V curves of D7Be-II together with the curves shown in Figure 5a,b. The reduction in capacitance in the PIN diodes is accompanied by a loss of linearity in the low-bias range [−15 V, 0 V] of the A^2^/C^2^ vs. V characteristics, as shown in Figure 5b, while the Schottky diodes retain a linear behavior. Figure 5c reports the doping profiles $N_{D}\;$ extracted from the C–V curves of the pristine devices. The extracted values are consistent with the nominal doping of the epitaxial layer. The depth axis represents the distance from the wafer surface: for Schottky diodes, it corresponds to the depletion width calculated from the Schottky model, whereas for PIN diodes, an offset of 0.7 µm is added to account for the position of the metallurgical junction below the surface. For the Be-implanted PIN diodes, the extraction of the doping profile from C–V measurements, either using the abrupt-junction approximation or the general capacitance expression, is not reliable. Since the Be implantation is directed only to the central region of the device area, the measured capacitance results from the parallel contribution of implanted and unimplanted regions. Consequently, a quantitative extraction of the doping profile from the overall capacitance was not reported in Figure 5c.

The effect of Be ion implantation on PIN diode capacitance was analyzed through numerical simulations performed with Synopsys Sentaurus TCAD, using the physical parameters reported in [31]. The simulated structure is one-dimensional and reproduces the experimental doping profiles. After calibration of the pristine device, Figure 5d compares the experimental C–V curves of D7Be-I with simulations obtained using a doping concentration of $1.05\times 10^{16}\;{\mathrm{cm}}^{-3}$, consistent with epitaxial growth tolerances, and an active area of $1.28\times 10^{-2}\;{\mathrm{cm}}^{2}$. The post-implantation C–V behavior is accurately reproduced by introducing a compensating negative charge distribution near the metallurgical junction, modeled with a Gaussian profile with 10^16^ cm^−3^ peak concentration, which reaches a concentration of 10^15^ cm^−3^ at a depth of approximately 4 µm.

## 4. Discussion

The reported electrical characterization aims to verify the feasibility of inducing and measuring a variation in the decay time of ^7^Be atoms implanted in the space-charge region of a reverse-biased PIN diode and the reliability of the ^7^Be decay time measurements, which depend on the value of the electric field and its stability over time.

I–V characterization of the pristine diodes (Figure 2 and Figure 3) was performed to evaluate the correspondence between the fabricated diode characteristics and the breakdown voltage calculated by TCAD simulations. In this work, breakdown was not reached in I–V measurements to avoid device damage, as the focus was on the operating region relevant for Stark effect generation. Instead, the maximum leakage current for reverse I–V characterization was set to 300 nA, which is a conservative value to avoid diode irreversible damage [23]. For each die, the diode with the lowest leakage current and the smallest current increase slope at 750 V reverse bias was selected for ^7^Be decay experiments. This selection is necessary because ^7^Be atoms must be confined within the electric field to experience the Stark effect and, therefore, must be implanted in a single device per die. Figure 3a shows that, among the eight dies tested at high reverse bias, all but one contain at least one diode with leakage current below 300 nA at 750 V. Figure 2 and Figure 3 also indicate that smaller-area diodes exhibit more uniform behavior, with clustered curves at both low and high voltages, but higher current densities in the [−110 V, 0 V] range (Figure 2b) and impact ionization occurring at lower bias with respect to the large area diodes (Figure 3a). These observations are attributed to perimeter currents [22] and to breakdown starting at the edge of the JTE (Figure 1b) since smaller diodes have a higher perimeter to area ratio. For these reasons, large-area diodes were selected for subsequent experiments.

Figure 4b shows that, after ^7^Be implantation, the leakage current increases in the diode that initially had low SRH current, while defect-induced carrier generation is negligible in the diode that already exhibited high SRH current in the pristine state. Under forward bias, Be implantation leads to an increase in the current “bump” and in the series resistance. These observations are consistent with the literature on 4H-SiC PiN diodes, where displacement damage (from irradiation or light-ion implantation such as Be) degrades forward I–V characteristics. This degradation is attributed to increased carrier recombination due to defects, compensation of the n-type region by acceptor centers, and reduced carrier mobility due to scattering [28,30]. The observed increase in reverse current after ^7^Be implantation is attributed to defect-induced generation centers within the bandgap. However, the blocking capability is preserved, as no premature abrupt rise indicative of impact ionization is observed within the measured voltage range.

The C–V characteristics (Figure 5a,b), and their interpretation (Figure 5d), support this picture. Several observations clarify the origin of the C–V modification: (i) loss of linearity in A^2^/C^2^–V curves is observed only in PIN diodes and not in Schottky diodes; (ii) this deviation appears already at low reverse bias, corresponding to depletion depths smaller than the projected Be range; (iii) the effect magnitude does not scale with implanted Be dose, nor with the ^7^Li contamination present in the ^7^Be implanted devices. These findings indicate that the capacitance variation is not directly due to Be atoms at their projected range, but rather to implantation-induced defects. The variation in C–V characteristics is attributed to acceptor-like defects generated by electronic stopping mechanisms, located near the surface and close to the p/n junction [30] or to nitrogen deactivation due to coupling with point defects [32]. In both cases, the net result is compensation of the donor doping and reduction in the effective free carrier concentration. Similar effects have been widely reported in SiC, especially after high-energy particle irradiation [28,33,34] or implantation of light ions such as He [30]. Flat C–V curves are typically observed in radiation detectors, where the epitaxial layer is lightly doped. In the present case, the epitaxial layer is more heavily doped, and the global dopant removal is therefore relatively small, explaining why no significant effect is observed in Schottky diodes. In contrast, in PIN diodes, the situation differs in the vicinity of the junction: here, the compensation induced by Be implantation-induced defects adds to the tail of the Al doping profile extending across the metallurgical junction, making the effective dopant removal locally non-negligible. Figure 5d shows that this results in a measurable reduction in capacitance, analogous to what is observed in low-doped epitaxial layers used for radiation detectors. Moving away from the junction, the compensation effect progressively weakens, and the apparent dopant concentration calculated from C–V approaches the nominal value.

**Figure 6:**
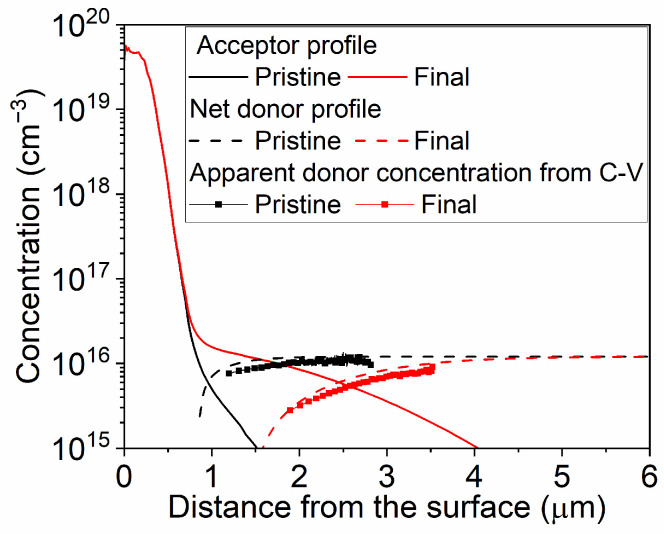
Apparent dopant profiles used to simulate the C–V curves of D7Be-I in the pristine (black lines) and final (red lines) stage compared to the apparent donor profiles extracted from the C–V curve analysis (black and red squares, respectively). The donor profiles obtained from measurements are shifted by the metallurgical junction position, either 0.7 µm or 1.4 µm.

Figure 6 reports the apparent dopant profiles, resulting from Al, acceptor defects, and epitaxial layer doping, used to simulate the C–V curves of D7Be-I in the pristine and in the final stage (black and blue lines, respectively). The black and blue symbols correspond to the apparent donor concentrations calculated from the C–V curves measured in the pristine and final stage, respectively. The profiles have been translated by 0.7 µm and 1.4 µm to account for the metallurgical junction position. Even if the Be implantation lowers the apparent donor profile in the epitaxial layer, operating at reverse bias exceeding the full depletion voltage of the PIN structure (~520 V) ensures that the Be-implanted region is fully depleted and immersed in an electric field comparable to that predicted by the simulations in Figure 1b.

**Figure 7:**
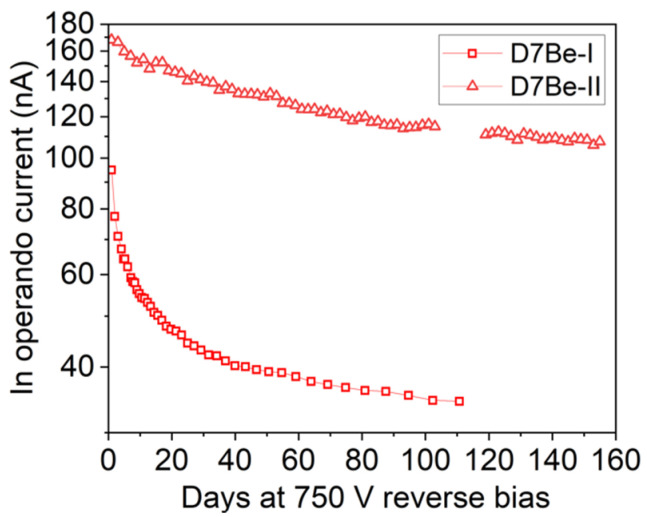
In operando current measured at 750 V reverse bias as a function of time in D7Be-I (open squares) and D7Be-II (open triangles).

Regarding stability, Figure 4b shows a decrease in current from the as-implanted to the final state. In operando current measurements (Figure 7) reveal that this decrease is gradual with a trend unrelated to the ^7^Be decay time, suggesting no direct connection with the decay process. Similar reductions in leakage current have been reported in SiC diodes after implantation or irradiation and are typically attributed to partial self-recovery of lattice damage or to surface leakage suppression induced by local heating under high reverse bias. Recently, a slight reduction in reverse current was measured in 4H-SiC diodes implanted with 5MeV ^7^Li [35]. All effects may occur here, but neither is expected to significantly alter the electric field in the space-charge region at high reverse bias; therefore, a definitive attribution would provide little value in this work.

Overall, these results indicate that the electric field experienced by ^7^Be remains stable and close to the expected value, as the most evident changes occur only after Be implantation, whereas both the reverse current and the extracted doping levels in the ^7^Be region do not show significant variation between the as-implanted and final stages.

## 5. Conclusions

We reported the design, fabrication, and electrical characterization of 4H-SiC PIN diodes suitable to provide a high electric field that can induce the Stark effect in ^7^Be atoms implanted in the space-charge region. Two devices were selected for the decay experiments, which involved undergoing ^7^Be ion implantation and 750 V continuous reverse bias for more than 100 days. I–V and C–V characterizations showed that ^7^Be implantation induces defects that increase the SRH recombination and compensate the donor doping close to the junction, causing an increase in diode series resistance and reduction in capacitance. However, in the working conditions of 750 V, the epitaxial layer is completely depleted, and the ^7^Be atoms are expected to be immersed in an electric field whose value is calculated to be about (1.3 ± 0.2) MV/cm. During the decay experiment, the reverse leakage current is mainly due to trap-assisted tunneling, with a slight decrease over time attributed to self-healing mechanisms. However, these are not affecting the electric field, proving the suitability of 4H-SiC diodes as a host material to probe the Stark effect, possibly affecting ^7^Be.

## Figures and Tables

**Table 1:** Summary of the experimental parameters employed both for the two PIN diodes used in the Stark effect experiment and of the test structures (one PIN diode and one Schottky diode): anode area, total area of the anode and JTE, Be implanted dose, Be depth and concentration calculated by SRIM, and duration of the long-term bias.

Name	Anode Area (cm^2^)	Total Area (cm^2^)	Be Fluence (cm^−2^)	Be Depth (µm)	Be Concentration (cm^−3^)	Bias (Days)
D7Be-I	9.88 × 10^−3^	1.28 × 10^−2^	1.6 × 10^12^	2.6–4.2	1 × 10^16^	107
D7Be-II	9.88 × 10^−3^	1.28 × 10^−2^	8 × 10^11^	3.0–3.8	1 × 10^16^	152
D9Be	7.85 × 10^−3^	1.07 × 10^−2^	1.33 × 10^12^	1.8–5.2	3.9 × 10^15^	30
S9Be	7.08 × 10^−3^	7.08 × 10^−3^	6 × 10^12^	1.4–2.0	1.0 × 10^17^	0

## Data Availability

The original contributions presented in this study are included in the article. Further inquiries can be directed to the corresponding author.

## Appendix A. Be Ion Implantation

Be ion implantation was carried out in selected devices, both PIN and Schottky diodes. While ^7^Be-implanted devices were employed for the ^7^Be decay experiments, tests were carried out by using the stable isotope ^9^Be. The implantation was carried out at the 3 MV Tandem accelerator laboratory of CIRCE-DMF [36,37] at the University of Campania “Luigi Vanvitelli.” A ^7^Be^2+^ beam was accelerated to the desired energy (2.5 to 4.2 MeV) and implanted into a SiC crystal placed in a vacuum chamber at room temperature. The crystal was oriented with its front face perpendicular to the beam axis to avoid the 4° channeling angle. The ^7^Be ions were produced using an MC-SNICS negative ion sputtering source. Briefly, ^7^Be was extracted from the cathode as a ^7^Be^16^O− molecular ion, selected by an electrostatic analyser and a dipole magnet, and then injected into the accelerator. The accelerator terminal, filled with argon gas, dissociated the molecular ion and produced positive charge states. The 2+ charge state was subsequently selected using an analyzing magnet and an electrostatic analyser to obtain the desired ^7^Be beam energy. The resulting beam was intrinsically contaminated by ^7^Li, which has the same mass number and therefore cannot be separated by the beam transport system. The level of contamination was determined using a silicon detector installed at the end of the implantation beamline and covered with a thin Mylar foil. After passing through the foil, the two isobars exhibited different residual energies, allowing them to be distinguished in the measured energy spectrum [17,38]. Table A1 reports, for each sample, the Be plateau concentration, the ^7^Li contamination in the ^7^Be implanted devices, the implantation energies, and the corresponding projected range calculated by SRIM for each device stack.

**Table A1:** ^7^Be plateau concentration and ^7^Li ratio in the ^7^Be implantation beam employed for D7Be-I and D7Be-II; ^9^Be plateau concentration in the ^9^Be implanted devices D9Be and S9Be; implantation energies and projected range calculated by SRIM for each device.

	D7Be-I		D7Be-II		D9Be		S9Be	
**[Be] (cm^−3^)**	1 × 10^16^		1 × 10^16^		3.9 × 10^15^		1 × 10^17^	
**^7^** **Li/(^7^Be + ^7^Li) (%)**	70–98		17–21		N.A		N.A.	
	**Energy (MeV)**	**R_p_** **(μm)**	**Energy (MeV)**	**R_p_** **(μm)**	**Energy (MeV)**	**R_p_** **(μm)**	**Energy (MeV)**	**R_p_** **(μm)**
	2.7	2.9	2.7	2.9	1.6	2	1.25	1.75
	3	3.1	3.2	3.3	2.3	2.7		
	3.5	3.6			2.95	3.2		
	3.9	4			3.6	3.8		
					4.3	4.5		
					4.9	5		

## Appendix B. C–V Characterization of D7Be-II

Appendix B reports the capacitance–voltage characterization carried out on D7Be-II. These are reported here for the sake of completeness since they yield similar results and information that can be drawn in the case of D7Be-I.
